# Clinical and linkage study on a consanguineous Chinese family with autosomal recessive high myopia

**Published:** 2009-02-09

**Authors:** Zhikuan Yang, Xueshan Xiao, Shiqiang Li, Qingjiong Zhang

**Affiliations:** State Key Laboratory of Ophthalmology, Zhongshan Ophthalmic Center, Sun Yat-sen University, Guangzhou, P. R. China

## Abstract

**Purpose:**

A linkage study on autosomal recessive high myopia (arHM) has not been reported, although several loci for autosomal dominant high myopia (adHM) have been mapped. Data from a consanguineous Chinese family with arHM were collected to map the genetic locus associated with this condition.

**Methods:**

Phenotypic information and DNA samples were collected from family members. A genome-wide linkage scan combined with homozygosity mapping was performed by using 382 microsatellite DNA markers from the entire genome spaced at intervals of about 10 cM.

**Results:**

The pedigree and clinical data of the family indicate that the high myopia is autosomal recessive. A genome-wide scan of chromosomes 1–22 gave a LOD score greater than 1.0 for 22 markers. Linkage to most of these markers was not supported by closely flanking markers except for three possible loci on chromosomes 11, 14, and 17. Fine mapping and haplotype analysis provide evidence for a locus at 14q22.1-q24.2 in a 25.23 Mb region between markers D14S984 and D14S999 with a maximum LOD score of 2.19. All 11 microsatellite markers inside the linkage interval as well as haplotype construction point to a gene at this locus. Linkage elsewhere on chromosome 11 and chromosome 17 could not be excluded due to the small size of the family.

**Conclusions:**

Pedigree and clinical data suggest that an autosomal recessive gene is responsible for high myopia in a consanguineous Chinese family. Genome-wide linkage analysis was used to map the gene for high myopia to a few limited loci. The resultant information should help future studies identify the gene for arHM. To our knowledge, this report is the first clinical and linkage study on a consanguineous family with arHM.

## Introduction

Myopia is a leading cause of visual impairment worldwide, and Chinese people living in industrialized or urban/suburban areas have a significantly higher incidence of myopia than other populations [1-4]. High myopia, its extreme form, is the fourth most common cause of irreversible blindness [5,6]. Evidence has shown that genetic factors play an important role in the development of high myopia [5,7-14].

High myopia may be inherited as an autosomal dominant, autosomal recessive, or X-linked recessive trait. Some high myopia could also be transmitted as a complex trait. Eight loci for autosomal dominant high myopia (adHM) and two loci for X-linked recessive high myopia (xlHM) have been mapped including myopia 1 (*MYP1* [Xq28]) [12,15], *MYP2* (18p11.31) [14,16], *MYP3* (12q21-q23) [13,17], *MYP4* (7q36) [18], *MYP5* (17q21-q22) [5], *MYP11* (4q22-q27) [10], *MYP12* (2q37.1) [11], *MYP13* (Xq23-q25) [9,19], and two others on 10q21.1 [8] and 5p15 [7] in which formal gene names have not yet been assigned. These loci, however, are estimated to be responsible for only a small portion of high myopia [20]. Although several candidate genes have been analyzed [21-26], the genes at these loci still remain unknown [21,27-29]. Furthermore, before this study, no genetic loci for autosomal recessive high myopia (arHM) have been reported. Identification of additional loci as well as of the causative genes is the first step toward understanding the molecular basis of myopia and, subsequently, toward the prevention and treatment of this sight threatening problem.

Here, we report a clinical and linkage study on a consanguineous Chinese family with arHM. A genome-wide linkage analysis combined with homozygosity mapping provides suggestive linkage of high myopia to a few loci including a novel locus in the q21-q24 region of chromosome 14 between markers D14S288 and D14S74.

## Methods

### Family and clinical data

**Figure 1:**
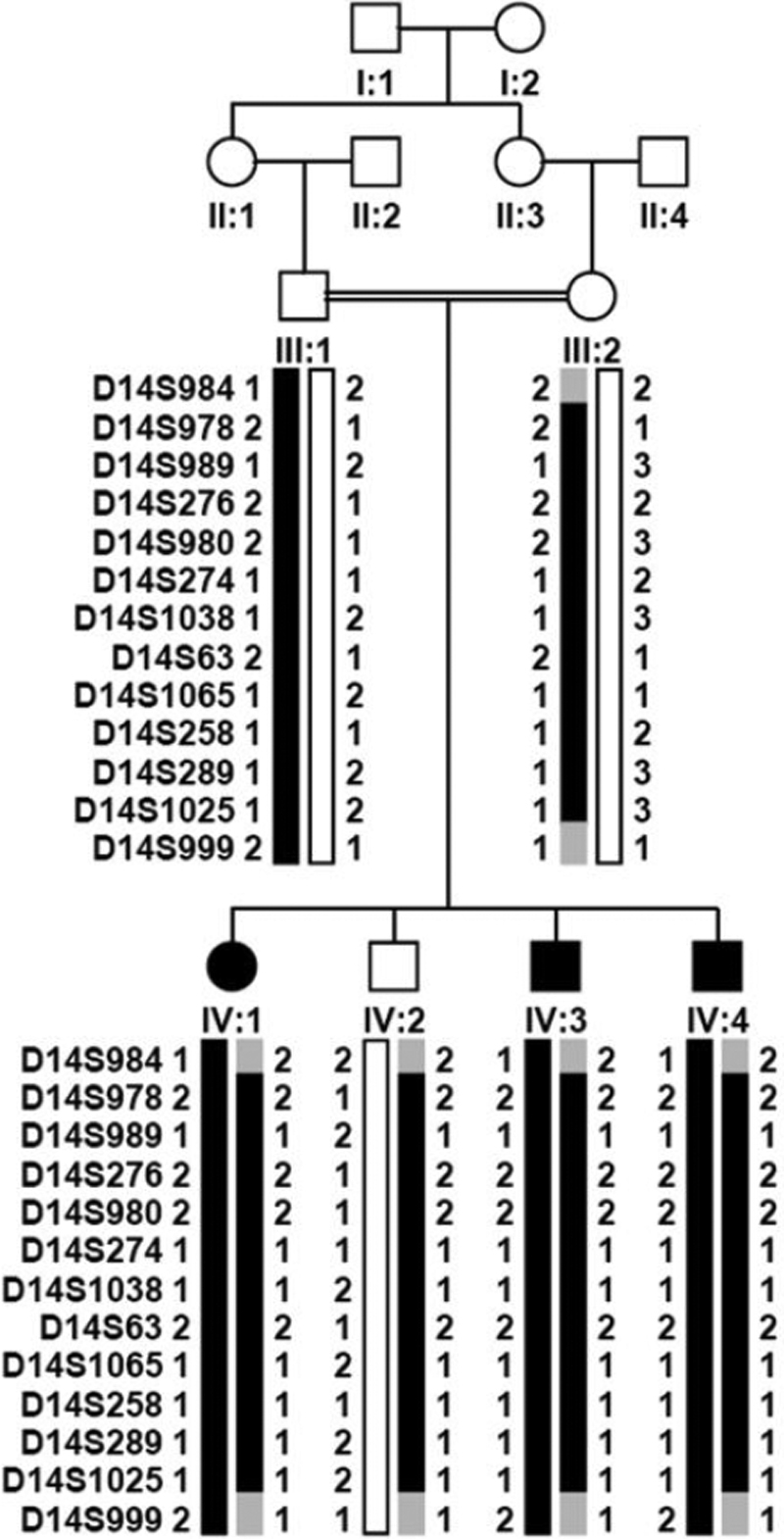
Pedigree and haplotype diagram of the family. Filled squares (male) or circles (female) represent individuals affected with high myopia. Blackened filled bars indicate the chromosomal regions that are derived from the ancestral disease-associated haplotype.

The consanguineous Chinese family with arHM was one of the 628 families with high myopia ascertained as part of a project to identify the genetic causes of high myopia in China. This family was from the Guangdong province of China. It includes one consanguineous marriage with three affected individuals (Figure 1). Three affected and three unaffected individuals from the family participated in this study. Informed consent conforming to the tenets of the Declaration of Helsinki and following the Guidance of Sample Collection of Human Genetic Diseases (863-Plan) by the Ministry of Public Health of China was obtained from the participating individuals before the study. Ophthalmological examination was performed at the Eye Hospital, Zhongshan Ophthalmic Center. Refractive error was measured by retinoscopy after mydriasis with compound tropicamide (Mydrin®-P; Santen Pharmaceutical Co. Ltd., Osaka, Japan). A subject was considered to have high myopia if he or she met the following criteria: 1) the myopia was noted before school age; 2a) cycloplegic refraction of −6.00 diopters (D) or lower (spherical equivalent) in individuals under 30 years of age or 2b) manifest refraction of −6.00 D or lower (spherical equivalent) in individuals 30 years old or older as defined previously [9]; and 3) exclusion of other known ocular or systemic diseases. Electroretinogram (ERG) responses were recorded in the proband consistent with the International Society for Clinical Electrophysiology of Vision (ISCEV) standards [30]. Genomic DNA was prepared from venous blood.

### Genotyping, linkage analysis, and candidate gene screening

Genotyping for all participating family members was performed using 5′-fluorescently labeled microsatellite markers as previously described [10], except that the amplicons were separated on an ABI 3100 Genetic Analyzer (Applied Biosystems, Foster City, CA). Genotyping data were analyzed using the Gene Mapper version 3.5 software package (Applied Biosystems). Two-point linkage analysis was performed by using the MLINK program of the FASTLINK implementation of the LINKAGE program package [31,32]. The myopia in the family was analyzed as an autosomal recessive trait with full penetrance and with a disease-gene allele frequency of 0.0001. Homozygosity mapping was used to exclude those markers with positive LOD scores but without homozygosity for alleles [33,34]. For fine mapping around the possible region, additional markers were selected according to the National Center for Biotechnology Information (NCBI) map. Haplotypes were generated using the Cyrillic 2.1 program (CyrillicSoftware, Wallingford, Oxfordshire OX10 8BA) and confirmed by inspection. The criteria for establishing linkage have been previously described [35].

## Results

**Figure 2:**
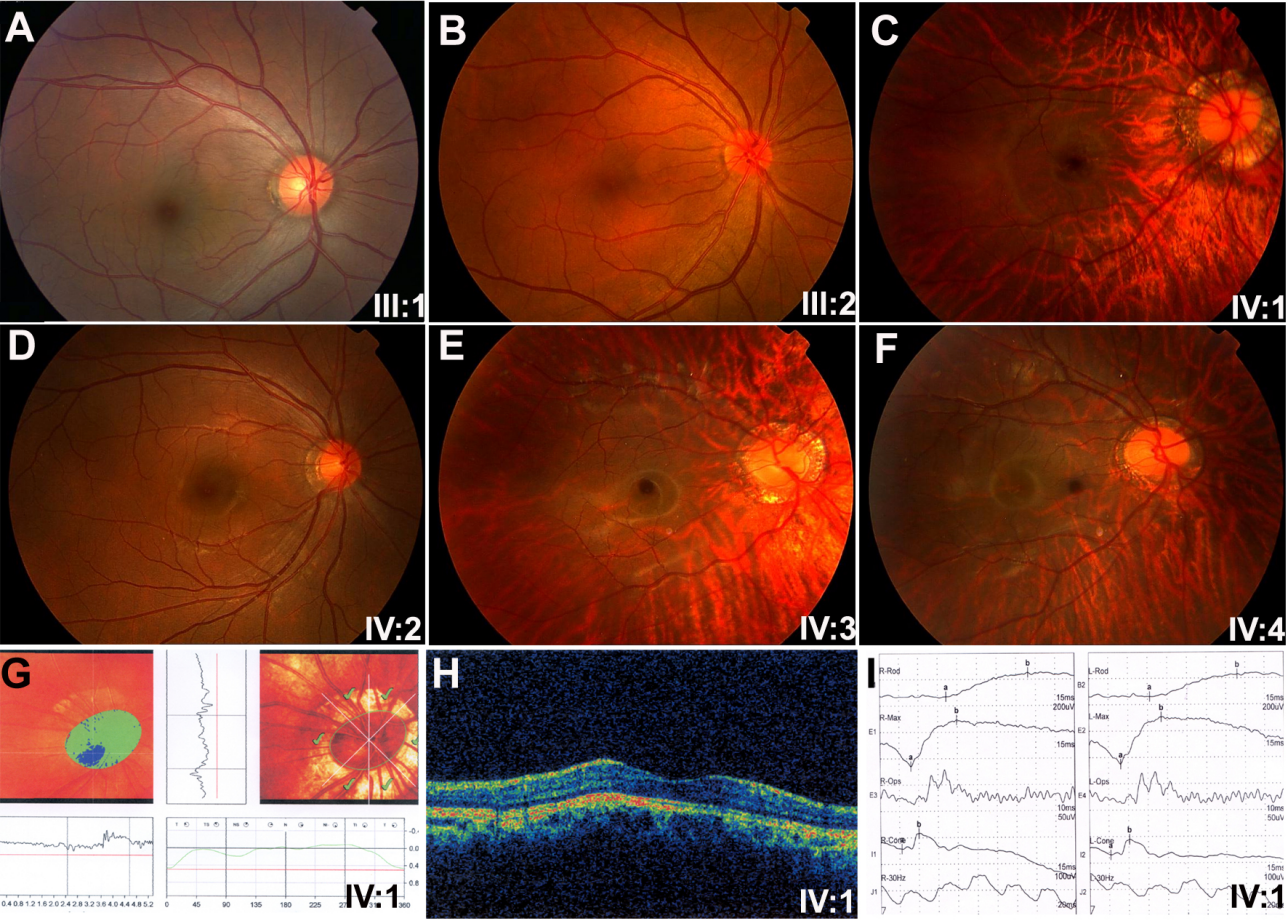
Clinical phenotypes of arHM. **A**-**F**: Fundus photos from parents (**A**, **B**) and affected (**C**, **E**, **F**) and unaffected (**D**) offspring. The individual numbers at the lower right corner of each photo are the same as those in Figure 1. All affected patients had a tigroid fundus change and circular choroidal defects around the optic discs. **G**-**I**: Results of HRT (**G**), OCT (**H**), and ERG (**I**) from affected individual IV:1 are shown. Normal HRT and OCT and mild reduced cone responses were shown.

The consanguineous family originates from a small town in northeast Guangdong province, which is about 400 km away from Guangzhou, China (Figure 1). Three of the four children have been affected with high myopia since early childhood. None of the affected siblings had night blindness or photophobia. Neither the parents nor the grandparents were affected. Ophthalmological examination revealed that all three affected siblings had normal corneas, irises, and lenses. All three affected children had extreme high myopia with excessive extension of the axial length (Figure 2, Table 1). Fundus observation of the three affected siblings demonstrated similar changes including tigroid fundus and a circular choroidal defect around the optic disc (Figure 2C,E,F). Optical coherence tomography (OCT) and a Heidelberg Retina Tomograph (HRT) examination revealed normal optic discs and normal thickness of the retinal layers (Figure 2G,H). The electroretinogram (ERG) records of two patients showed a mild reduced amplitude of the cone response (Figure 2I). All patients had normal color vision based on Ishihara plate screening. No systemic abnormalities were noted in the affected individuals.

**Table 1:** Clinical data for the family with autosomal recessive high myopia.

**ID**	**Age at onset (years)**	**Age at exam (years)**	**Sex**	**Unaided best visual acuity (corrected)**		**Refraction**		**Axial length (mm)**		**Fundus**
				**OD**	**OS**	**OD**	**OS**	**OD**	**OS**	
III:1		47	M	1.5	1.2	plano	-0.25DCx45	23.17	23.12	Normal
III:2		46	F	0.8	0.7	-1.50DS+2.25DCx170	-1.25DCx90	22.59	22.50	Normal
VI:1	Early childhood	20	F	0.03 (0.8)	0.05 (0.8)	-12.00DS-2.50DCx180	-10.50DS-2.50DCx10	27.29	26.86	Myopic
VI:2	16	19	M	0.1 (1.0)	1.0	-2.00DS	plano	23.96	22.85	Normal
VI:3	Early childhood	17	M	0.1 (1.0)	0.2 (1.0)	-15.50DS-2.50DCx15	-12.50DS-3.50DCx170	29.4	28.24	Myopic
VI:4	Early childhood	16	M	0.02 (0.8)	0.02 (0.8)	-13.00DS-4.00DCx10	-14.00DS-5.00DCx170	28.9	29.16	Myopic

All three affected individuals had high myopia since early childhood. They had extreme high myopia at examination with apparent elongation of the axial length of both eyes. In the table, “plano” indicates no refractive error or zero.

Linkage to all known loci for high myopia was initially excluded. A genome-wide scan excluded linkage to 313 of the 382 panel markers for chromosomes 1−22 by a LOD score equal to or lower than −2.0. Twenty markers yielding LOD scores between -2.0 and 0 were further excluded from linkage due to heterozygous alleles by homozygosity mapping. For the remaining 49 markers with LOD scores greater than −2.0, markers D7S513 and D12S310 were unsuccessful for genotyping, and 25 of these markers with positive LOD scores of less than 1.0 were not informative where the neighboring markers gave LOD scores of less than −2.0.

Only 22 markers yielded two-point LOD scores greater than 1.0 in a genome-wide scan of chromosomes 1−22 (Table 2). Linkage to most of these markers was not supported by closely flanking markers except for three loci on chromosomes 11, 14, and 17. Fine mapping and haplotype analysis provide strong evidence for a locus on chromosome 14q22.1-q24.2 (Figure 1, Table 3). This locus maps to a 25.23 Mb region between D14S984 and D14S999 with maximum LOD scores of 2.19 at θ=0 for D14S989, D14S980, D14S1038, D14S289, and D14S1025, reaching the theoretical maximum LOD score that could be generated in this type of family. All 11 microsatellite markers examined inside the linkage interval and the haplotype construction of these markers support this locus. However, linkage to regions of chromosomes 11 and 17 could not be excluded due to the small size of the family.

**Table 2:** Markers yielding a two-point LOD score over 1.0 from a genome-wide scan.

**Markers**	**LOD score at θ=**						
	**0.00**	**0.01**	**0.05**	**0.10**	**0.20**	**0.30**	**0.40**
D1S199	1.43	1.39	1.27	1.11	0.80	0.49	0.22
D3S1580	2.03	1.99	1.82	1.60	1.14	0.67	0.23
D4S406	1.12	1.10	1.00	0.88	0.62	0.38	0.17
D4S1597	1.43	1.39	1.27	1.11	0.80	0.49	0.22
D7S657	2.19	2.14	1.96	1.72	1.24	0.77	0.33
D9S286	1.43	1.39	1.27	1.11	0.80	0.49	0.22
D10S249	1.12	1.10	1.00	0.88	0.62	0.38	0.17
D10S189	2.03	1.98	1.8	1.56	1.08	0.61	0.19
**D11S901**	1.12	1.10	1.00	0.88	0.62	0.38	0.17
**D11S898**	1.43	1.39	1.27	1.11	0.80	0.49	0.22
**D11S1320**	1.43	1.39	1.27	1.11	0.80	0.49	0.22
**D11S968**	1.43	1.39	1.27	1.11	0.80	0.49	0.22
D13S265	1.12	1.10	1.00	0.88	0.62	0.38	0.17
**D14S276**	1.43	1.39	1.27	1.11	0.80	0.49	0.22
**D14S63**	2.03	1.98	1.8	1.56	1.08	0.61	0.19
**D14S258**	1.12	1.10	1.00	0.88	0.62	0.38	0.17
D16S404	1.12	1.10	1.00	0.88	0.62	0.38	0.17
D16S3046	1.12	1.10	1.00	0.88	0.62	0.38	0.17
**D17S1868**	1.12	1.10	1.00	0.88	0.62	0.38	0.17
**D17S787**	2.19	2.15	1.98	1.76	1.30	0.83	0.37
D18S452	1.43	1.39	1.27	1.11	0.80	0.49	0.22
D19S571	1.43	1.39	1.27	1.11	0.80	0.49	0.22

It is common to have several dozen of markers with positive LOD scores in a genome-wide scan in a family of moderate size. A single marker with a LOD score over 1.0 is usually not an indication of suggestive linkage if it is not supported by closely flanking markers. To report all markers with positive LOD scores greater than 1.0 may provide useful clues for subsequent linkage study of other families with arHM. Markers on relevant regions of chromosomes 11, 14, and 17 are highlighted in bold.

**Table 3:** Two-point linkage results for markers at 14q22.1-q24.2.

**Markers**	**Position**		**LOD score at θ=**						
	cM*	**Mb#**	**0.00**	**0.01**	**0.05**	**0.10**	**0.20**	**0.30**	**0.40**
D14S984	43.60	49.17	−2.28	−0.97	−0.39	−0.2	−0.13	−0.15	−0.13
D14S978	44.20	50.98	2.03	1.98	1.80	1.56	1.08	0.61	0.19
D14S989	46.20	52.77	2.19	2.14	1.96	1.72	1.24	0.77	0.33
D14S276	47.00	54.75	1.43	1.39	1.27	1.11	0.80	0.49	0.22
D14S980	50.90	56.22	2.19	2.14	1.96	1.72	1.24	0.77	0.33
D14S274	53.80	56.73	1.12	1.10	1.00	0.88	0.62	0.38	0.17
D14S1038	56.70	58.69	2.19	2.14	1.96	1.72	1.24	0.77	0.33
D14S63	59.00	63.72	2.03	1.98	1.80	1.56	1.08	0.61	0.19
D14S1065	63.00	67.98	1.43	1.39	1.27	1.11	0.80	0.49	0.22
D14S258	65.80	69.65	1.12	1.10	1.00	0.88	0.62	0.38	0.17
D14S289	67.10	70.63	2.19	2.14	1.96	1.72	1.24	0.77	0.33
D14S1025	71.60	73.31	2.19	2.14	1.96	1.72	1.24	0.77	0.33
D14S999	73.70	74.40	−2.28	−0.97	−0.39	−0.2	−0.13	−0.15	−0.13

Fine mapping demonstrates strong evidence of suggestive linkage for a novel arHM locus on chromosome 14q22.1-q24.2. This locus encompasses a 25.23 Mb region between D14S984 and D14S999. All 11 microsatellite markers examined inside the linkage interval support this locus by yielding positive LOD scores, with maximum LOD scores of 2.19 at θ=0 for D14S989, D14S980, D14S1038, D14S289, and D14S1025. The asterisk indicates the genetic position (cM, centimorgan) of each marker based on data from Genethon; The sharp (hash mark) indicates the physical position (Mb, mega base pairs) of each marker according to the database of Homosapiens Build 36.3.

## Discussion

In this study, we described a consanguineous Chinese family with three offspring affected with high myopia. All three affected family members presented with extreme high myopia in early childhood, which is characterized by a typical and similar myopic fundus and an obvious elongation of ocular axial length. Other ocular and systemic diseases were excluded. These similar characteristics together with pedigree and family information such as the comparatively normal refraction and fundus in parents suggest that the high myopia in this family is inherited as an autosomal recessive trait. Although arHM has been suggested by population and segregation analysis [36-38], arHM in any consanguineous family has not been previously reported with accompanying clinical and linkage data.

It has been suggested that arHM represents a common form of hereditary high myopia [36-39]. Unfortunately, linkage studies on this type of high myopia have been lacking. Of the 10 loci that have been previously mapped for high myopia, eight are for adHM and the two others for xlHM. None were for arHM. Two major problems may contribute to the difficulty in identifying the exact loci and genes for high myopia [10]. First, are we really able to differentiate hereditary high myopia from acquired high myopia? That is, do we determine the affected and unaffected status correctly in linkage analyses? Second, as high myopia is very common, the introduction of another myopia-related gene into a family such as by marriage can confound the identification of loci and genes. Therefore, a high myopia family with clearly defined phenotypes and minimum environmental impact would be ideal for finding the responsible locus or gene. The family reported in this study has typical hereditary high myopia and a clearly defined phenotype without any overlapping signs with unaffected individuals. This study, to our knowledge, is the first genome-wide linkage study performed for arHM. We have mapped the gene for arHM to a few limited loci, especially a 25.23 Mb region between D14S984 and D14S999 at chromosome 14q22.1-q24.2. Potential candidate genes in this linkage interval may include but are not limited to guanine nucleotide binding protein (G protein), gamma 2 (*GNG2*), G protein-coupled receptor 135 (*GPR135*), SIX homeobox 4 (*SIX4*), and regulator of G-protein signaling 6 (*RGS6*). Even though the exact locus and gene have not been defined, our study does provide useful clues for identifying the gene for arHM in further studies. Recruitment of additional families could possibly help to define the exact locus, which would lead to the cloning of the causative gene.
